# DOxy: A Dissolved Oxygen Monitoring System

**DOI:** 10.3390/s24103253

**Published:** 2024-05-20

**Authors:** Navid Shaghaghi, Frankie Fazlollahi, Tushar Shrivastav, Adam Graham, Jesse Mayer, Brian Liu, Gavin Jiang, Naveen Govindaraju, Sparsh Garg, Katherine Dunigan, Peter Ferguson

**Affiliations:** Ethical, Pragmatic, and Intelligent Computing (EPIC) Research Laboratory, Department of Computer Science and Engineering (CSEN), School of Engineering (SoE), Santa Clara University (SCU), Santa Clara, CA 95053, USA

**Keywords:** Aquaculture Technology, Dissolved Oxygen (DO) Monitoring, Internet of Things (IoT), Pragmatic Resource Optimization (PRO), Sustainable Automation, Water Quality Testing

## Abstract

Dissolved Oxygen (DO) in water enables marine life. Measuring the prevalence of DO in a body of water is an important part of sustainability efforts because low oxygen levels are a primary indicator of contamination and distress in bodies of water. Therefore, aquariums and aquaculture of all types are in need of near real-time dissolved oxygen monitoring and spend a lot of money on purchasing and maintaining DO meters that are either expensive, inefficient, or manually operated—in which case they also need to ensure that manual readings are taken frequently which is time consuming. Hence a cost-effective and sustainable automated Internet of Things (IoT) system for this task is necessary and long overdue. DOxy, is such an IoT system under research and development at Santa Clara University’s Ethical, Pragmatic, and Intelligent Computing (EPIC) Laboratory which utilizes cost-effective, accessible, and sustainable Sensing Units (SUs) for measuring the dissolved oxygen levels present in bodies of water which send their readings to a web based cloud infrastructure for storage, analysis, and visualization. DOxy’s SUs are equipped with a High-sensitivity Pulse Oximeter meant for measuring dissolved oxygen levels in human blood, not water. Hence a number of parallel readings of water samples were gathered by both the High-sensitivity Pulse Oximeter and a standard dissolved oxygen meter. Then, two approaches for relating the readings were investigated. In the first, various machine learning models were trained and tested to produce a dynamic mapping of sensor readings to actual DO values. In the second, curve-fitting models were used to produce a successful conversion formula usable in the DOxy SUs offline. Both proved successful in producing accurate results.

## 1. Introduction

Oxygen from the atmosphere dissolves into rivers, lakes, and oceans and is consumed by aquatic animals for respiration [1]. Dissolved oxygen (DO) is hence considered to be the most important variable in water quality as marine life will suffocate if its concentration in water gets too low. Therefore, aquaculture industries monitor the water circulating through their systems as even slight changes in water quality can have severe negative effects on their crops. For instance, poor oxygen management in aquaculture systems can lead to physiological damage and substandard growth in the aquatic organisms being cultured [2,3,4] with most organisms sustaining damage when their ambient concentration of dissolved oxygen drops below ∼5% [5]. As such, proper management of DO levels is imperative and requires diligence in taking DO measurements.

Another reason why DO is such a critical environmental variable is how dynamic it is: over a matter of hours or even only minutes, dissolved oxygen levels can change from optimal to lethal [6] Therefore, since the response time for taking corrective measures is typically short, it is necessary to have a rapid and reliable method of continuously monitoring DO concentrations so that water facilitators can be proactive in improving the water’s quality [6]. There are numerous issues with the current standard methods of measuring DO in water though, including affordability, maintainability, and environmental safety—especially with chemical-based meters. Thus, research was conducted on the use of infrared technology as a means to measure DO in water and it was found that infrared sensors were capable of performing this task while addressing the lack of affordability, difficulty of maintenance, and potential environmental safety issues with the current standard of measuring methods. The preliminary findings were reported in a short 2020 paper with the same title that was presented at the 2020 IEEE Global Humanitarian Technology Conference (GHTC) and published as part of its proceedings [7]. This paper, in part, serves as an extended version of that paper but goes far beyond it.

Section 2 details existing methodologies for measuring dissolved oxygen and Section 3 reports on existing research and commercial products. Section 4 delineates how DO was measured in this research as well as how the sensors were calibrated. Section 5 provides the technical setup of the DOxy hardware and software followed by Section 6 which reports on the results from DOxy’s field testing. Finally, Section 7 and Section 8 respectively provide the current work in progress by the team and some closing remarks.

## 2. Methodologies for Dissolved Oxygen Sensing

Two general methodologies for measuring dissolved oxygen in water exist: Electrochemical and Optical. A Short explanations of the two and the issues associated with each follow below.

### 2.1. Electrochemical

#### 2.1.1. Methodology

There are two types of electrochemical dissolved oxygen sensors: galvanic and polarographic. Both methods utilize two polarized electrodes with differences in reactivity in an inert electrolyte solution that is not part of the reaction. A semi-permeable membrane separates the electrodes and the electrolyte solution from which oxygen diffuses across. dissolved oxygen is reduced at the cathode which causes an electrical current that is carried by the ions in the electrolyte to the anode. The measured electrical current provides information on the concentration of dissolved oxygen due to their direct relation [8]. Both methods work in a similar manner except for that in the galvanic method, there is no need to allocate warm-up time due to the self-polarization of the dissimilar metals used as the anode and cathode, such as zinc and silver. However, in the polarographic method, warm-up time is essential to polarize the electrodes as the metals used, such as gold and silver, do not have a large difference in reactivity [8].

#### 2.1.2. Problems

Although both electrochemical methods have advantages and can return a result quickly, there are a number of inconveniences encountered. Since the electrodes employed in both methods consume oxygen, the electrochemical method requires constant maintenance and thus recalibration every two to eight weeks and thus introduces a high maintenance cost while reducing efficiency and reliability, thus making it problematic to employ over sustained intervals [9]. For the polarographic electrochemical method specifically, the electrolyte needs to be replaced, and in the galvanic electrochemical method, the anode needs to be replaced as they are used up in the internal reactions [10]. This results in costly sensors with a short lifespan. Furthermore, the measurement accuracy of these electrochemical sensors may be lowered due to interference by certain chemical compounds such as hydrogen sulfide found in some bodies of water that may infiltrate the membrane.

### 2.2. Optical

#### 2.2.1. Methodology

The set up of an optical sensor consists of a semi-permeable membrane, a sensing element, a light-emitting diode, and a photodetector. The sensing element contains a luminescent dye that is immobilized in sol-gel. The dye becomes excited and emits light when exposed to the blue light emitted by the LED in the presence of DO [8]. The intensity and luminescence of the dye when exposed to blue light and the wavelength of the emitted light is dependent on the amount of dissolved oxygen in the water sample. The intensity of the returned luminescence is measured by a photodetector and is used to calculate the dissolved oxygen concentration [8].

#### 2.2.2. Problems

Optical dissolved oxygen sensors usually require more power and take 2–4 times longer to take a measurement than the electrochemical method [8]. These sensors are also heavily dependent on ambient conditions because of the luminescent dye’s sensitivity to temperature. Additionally, the luminescent dye eventually degrades. To maintain this type of sensor, one or two calibrations per year and a replacement cap every 18 months is needed [11]. Although the optical sensor has a lower maintenance cost, it has a greater acquisition cost which fish farmers and others small producers in the aquaculture industry may not be able to easily afford.

## 3. Related Works

### 3.1. Academic Research

#### 3.1.1. Utilization of Light Waves for Measuring DO in Water

Concerned with the use of nonrenewable transition metal complexes in quenching DO sensors, Silva et al. [12] derived transition metal complexes from kale using extraction, acidification, and complexation techniques to target chlorophyll A molecules in the kale and substitute magnesium with zinc ions. The extracted transition metal chlorophyll-zinc complexes were immobilized in a thin film of sol-gel, as per standard procedure for the construction of quenching DO complexes. The thin film was put over the surface of a sample of water and an LED emitted blue light at it which was then detected by a photodiode. The characteristic wavelength of chlorophyll-zinc complexes was measured to be 635 nm. When the DO concentration was made homogeneous across the analyte with a stirrer, the R^2^ of the zinc chlorophyll complexes, when were used for DO sensing, was 0.98282.

In a different approach, Zhao et al. used light in fluorescence quenching to measure DO [13] by coating an optical fiber with a fluorophore, Trisaminomethane Ruthenium (II) Complex Dichloride, that quenched in the presence of DO. Light was sent down the fiber and the returning light was measured and used to derive the partial pressure of DO in the solution using the stern-volmer equation. To compensate for the brightness fluctuation inherent in the light source, the team coated the tip of the optic fiber with CdSe/ZnS quantum dots, allowing them to quantize the fluctuation and to calibrate their results. The collected data, when regressed onto the line predicted by the stern-volmer equation, was able to achieve an R^2^ of 0.9957.

In order to enable the measurement of even lower levels of DO in water, Yu et al. [14] created a quenching-based DO sensor specifically for the measurement of DO in the range of 1 μm and below. Since they were also concerned with the common use of transition metal complexes in DO sensing due to their high cost and toxicity, Yu et al. chose to use metal-free organic phosphors, which, just like transition metal complexes, have long decay times. A challenge was the inundation of room temperature fluorescence in metal-free organic phosphors, since this requires particular conditions. The team ended up creating shell-core nanoparticles. The core was comprised of the metal-free phosphors embedded in a matrix of polystyrene (chosen because of its oxygen permeability). Poly(2-Methyl-2-Oxazoline) was chosen as the outer shell for its water solubility and biocompatibility. Upon testing, the nanoparticles were found to be particularly sensitive to the presence of oxygen. When dispersed within water containing DO and exposed to UV light, the nanoparticles exhibited very little fluorescence. As the water was sparged with nitrogen, the nanoparticles gradually became more fluorescent.

More broadly, Miura et al. [15] studied the absorbance levels of DO for different wavelengths. Tests were conducted on both tap water and seawater, with samples from each type of water brought to 100% DO through aeration and 0% DO through reduction by sodium-sulfate. From the 108 samples of sea water and tap water tested on, they found that the greatest variance in absorbance occurred in the blue and infrared wavelengths of light and likely to be most detectable under blue light. The researchers then built a prototype sensor and conducted three tests on it. In the first test, both the LED and the photodiode were exposed to saline water. The researchers found that in this case, contact with the saline water significantly effected experimental results. For the second and third tests, one component was kept dry while the other was exposed to saline water. In the second and third tests, where only the LED and only the photodiode were exposed to saline water respectively, only a small effect on the results was noted. The researchers thus concluded that it was important to separate the water being measured from touching the sensor.

#### 3.1.2. Use of Machine Learning in Calibration of DO Sensing

Zhang et al. [16] investigated the use of machine learning in the calibration of DO sensors. The team built an apparatus for sensor calibration, a chamber filled with ultrapure water in which an oxygen sensor, to be calibrated, and a reference sensor were suspended. The design of the apparatus allowed for the control of the DO concentration, the salinity, the temperature, and the pressure within the chamber. A small tube brings water from the chamber as an analyte for winkler iodometric titration analysis in order to allow for constant monitoring of the DO concentration. The collected data is then fed to a backpropagation neural network, which is run for a thousand iterations. When the calibrated model was finished, the team compared the accuracy of a quenching DO sensor to that of winkler analysis. The R^2^ of the model was 0.99971, while the R^2^ of traditional winkler analysis was 0.99839. When its performance was compared with that of an Anderaa sensor, the quenching DO sensor was found to produce results that were reliably similar.

In a different approach, Michelucci et al. [17] applied machine learning directly to the quenching O_2_ sensing. Typically, the magnitude of the quenching is related to O_2_ concentration with the stern-volmer equation which needs precisely calibrated sensors at one or more known concentrations, but the team instead trained a machine learning model to notice the correlation between the sensor inputs and O_2_ concentration values. The team utilized a commercial Pt-TFPP quenching-based sensor in a thermally controlled chamber in which the O_2_ concentration could be varied and homogenized across the chamber. The authors used a feedforward neural network to relate several variables within the chamber to the O_2_ concentration within it. Because of the paucity of extensive empirical datasets with respect to dissolved oxygen sensing, the team was forced to procedurally generate data with theoretical knowledge. The neural network was ran for three different network architectures and the mean absolute error (MAE) was calculated over each of them as they were given more layers and neurons, thus increasing in complexity. Two of the neural networks approached a minimum MAE of 0.012% on account of most of the data being theoretically generated. The third network stabilized at an MAE of 0.5%.

#### 3.1.3. IoT DO Sensing

Yunfeng et al. [18] noticed a trend in oxygen-controlling equipment for aquaculture: they were black boxes that did not transmit data to their users. To remedy this, the team developed an IoT platform to support the real-time monitoring of DO levels in fish ponds. The team deployed several sensors with each unit endowed with an electrochemical DO sensors, a temperature sensor, and a narrowband sending system. The device transmits data at intervals before returning to low-power mode.

In a much more sophisticated, yet still electromechanical DO Sensing approach, Stine et al. [19] developed a system for gathering real-time data on the topology of oxygen distribution within a bioreactor. To achieve this they designed DO-measuring IoT sensors to be dispersed through the aqueous contents of a bioreactor. The sensors were designed to contain electrochemical DO detectors. Each sensor contained a potassium chloride electrolyte with a thin-film gold working electrode, a gold counter electrode, and a silver reference electrode. The researchers communicated with the device network using a smartphone app, Silicon Labs, which allowed them to turn all the devices in the network to a low energy setting, calibrate the devices, and had the devices make intermittent measurements. A pod was placed in a 10 L bioreactor filled with De-ionized (DI) water in order to test it. Oxygen and nitrogen gas were pumped into the DI water before it was stirred with an impeller. A cyclic voltammogram found that cathodic current was maximized when the potential was between −0.4 and −0.6 V. Using a value in this range, −0.42 V, the researchers used chronoamperometry to determine that there was an average difference of 2.5 μA in current between when the solution was diluted with gas and when it was sparged with nitrogen gas. Tethering the pod to a 3.3 V power supply, the researchers calibrated the pod on −0.5 V every 30 s at several different oxygen levels. Using this data, the researchers were able to obtain a calibration plot mapping the oxygen levels in the solution to the voltage outputted by the pod. The correlation between the two quantities was found to have an R^2^ of 0.98, a sensitivity of 37.5 nA/DO%, and a limit of detection of 8.26 DO%. Finally, the researchers tested the pod while the bioreactor ran a fermenter in order to more closely simulate a working environment. The voltage outputted by the pod was found to increase linearly and inversely proportionally with DO% concentration, showing that it had been successfully implemented. Some problems still remained, however. For the first 45 min of the test, the difference between measurements by the pod and a polarographic DO sensor, which had been introduced into the bioreactor as a control, was less than four percent. As the bioreactor continued to operate, however, the difference began to shift, which the researchers suspected was due to the degradation of the reference electrode after continual usage. In response the researchers fitted the sensors with several design improvements. Since the inaccuracy grew linearly with time, they applied a correction factor to multiply the pod output with in order to refactor the results back down to under the four percent range.

Using a completely different approach, Hu et al. [20] observed that many aquaculturists monitored DO levels by observing fish behavior rather than using sensors. Even though this approach seemed cheaper it could only be done after the fish had been affected by deleteriously low levels of DO. To remedy this, they engineered a Radial Basis Function Neural Network and trained it on data from aquaculture ponds in Zhenjiang, China so that it could observe and thus predict the trend of the DO ahead of time. To optimize the Neural Network, a genetic algorithm was run on it. When compared with real data, the model showed high prediction accuracy.

### 3.2. Existing Products

Currently, the existing dissolved oxygen meters on the market are expensive, have high maintenance costs, or do not have wireless communication integrated into the device to enable continued remote monitoring of the dissolved oxygen levels.

For instance, Cole-Parmer which is a well known scientific and industrial instrument distributor has an array of costly dissolved oxygen Meters ranging from $265 to $2459 [21] at the time of this writing. Additionally, although their products have advantages such as features that allow calibration and measurement data to be stored with a timestamp, the meters have high maintenance costs due to replacements of the chemical solutions, membranes, and caps of the measurement probes. And most importantly, the devices offered are handheld and do not provide continuous monitoring in real time.

A similar company, Hanna Instruments, offers dissolved oxygen monitors with costs ranging from $220 to $1450 [22] at the time of this writing. Their products are also high maintenance as the solutions, membranes, and probe caps need to be replaced. Because wireless communication is not offered, testing on site is required and the device can not provide continuous monitoring.

Such devices that do not include long range wireless communication, hinder fish farmers from having the ability to detect variations in oxygen levels instantly and continuously. Therefore the farmers need to manually measure the dissolved oxygen levels several times per day which increases the amount of manual labor and reduces efficiency. Manual measurements are not only time consuming, they may also be inaccurate especially if the meters used are not calibrated and maintained correctly. Another advantage of an automated system would be the continues calibration and monitoring of individual sensors within the system which can also alert users to the malfunctioning of a sensor and or even diagnose the problem with the sensor(s) so that the technicians can repair or replace the faulty sensor(s).

## 4. Measuring Dissolved Oxygen (DO)

### 4.1. Data Collection

Various water solutions of differing dissolved oxygen levels were created and used to gather IR readings using a Maxim Integrated MAX30102 (San Jose, CA, USA), High-Sensitivity Pulse Oximeter sensor which has a red light wave range sensitivity of 650–670 nm and an IR light wave range sensitivity of 870–900 nm [23]. The sensor is capable of sampling received red/IR light waves in a 50 to 3200 samples per second (sps) range and reports the measured range in terms of h1Analog to Digital Converter (ADC) counts which are a count of how many Samples were detected per one second. The solution concentrations were selected in a random order in order to avoid bias in the readings due to time. A Milwaukee MW600 Portable Dissolved Oxygen Meter (Rocky Mount, NC, USA) with a DO measuring range of 0.0 to 19.9 mg/L [24] was used to measure the dissolved oxygen content of each water solution in parallel in order to equate the IR and DO readings.

#### 4.1.1. Water Sample Creation

At first, various local tap, bottled water, and deionized water supplies were used. After some experimentation, deionized water was determined to perform the best due to being free of any substances that could absorb the infrared wave of the Oximeter and thus result in inaccurate readings. A zero dissolved oxygen solution was created by mixing deionized water with sodium sulfite as it acts as an ‘oxygen scavenger’. Sodium sulfite reacts with the dissolved oxygen and forms sodium sulfate as seen in Equation (1), which does not affect readings of the infrared sensor.

(1) $$2Na_{2}SO_{3}+O_{2}⟶2NaSO_{4}$$

Thus, multiple different samples between 0% and 100% were created by simply using the ratio of 2 molecules of sodium sulfite to 1 molecule of dissolved oxygen. Furthermore, as the dissolved oxygen content of the solution increased gradually by being exposed to the open air over the course of ten days at room temperature, more readings were taken at various intervals by both the Maxim Integrated MAX30102 sensor and the Milwaukee MW600 meter.

#### 4.1.2. Data Gathering Process

**Figure 1:**
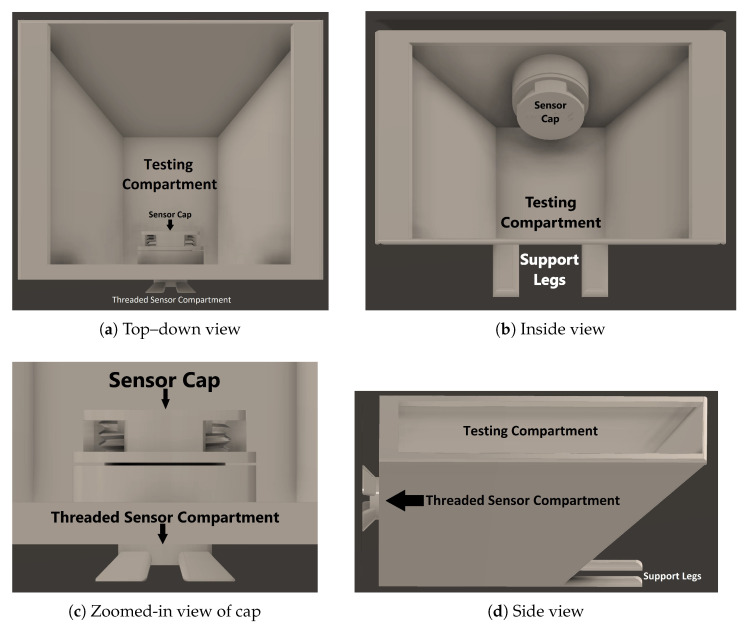
DOxy testing setup.

The Maxim Integrated MAX30102 High-Sensitivity Pulse Oximeter sensor is not waterproof. Hence, thin plexiglass [25] with a thickness of 1/32 of an inch was utilized to create a barrier between the sensor and water samples. To hold the sensor and plexiglass firmly together, a 3D-printed apparatus was designed and utilized, as can be seen in Figure 1.

The sensor is attached to a threaded component that screws into the plexiglass component so that the sensor would not move while gathering readings, and to prevent any air from being trapped in between the sensor and the plexiglass. Then one of the water solutions was poured into the pale until the water completely submerged the plexiglass chamber, ensuring that the sensor’s light would be solely transmitted through the plexiglass and water. Next the 2.5 mm cap was screwed on the side of the plexiglass, to create a chamber with a set distance for the sensor’s light to reflect back from. Screwing in the cap after the water was set ensured that there were no air bubbles in the chamber to interfere with readings. Lastly, the pale was covered in order to prevent any interference from ambient light and IR. Then, the red LED and infrared ADC counts (which are the number of red/IR samples recorded by the sensor’s photodiod per each second) were collected.

This method was used for each of the water solutions with differing dissolved oxygen content and compiled into a spreadsheet to be used for analysis and regression.

### 4.2. Data Analysis and Machine Learning

**Figure 2:**
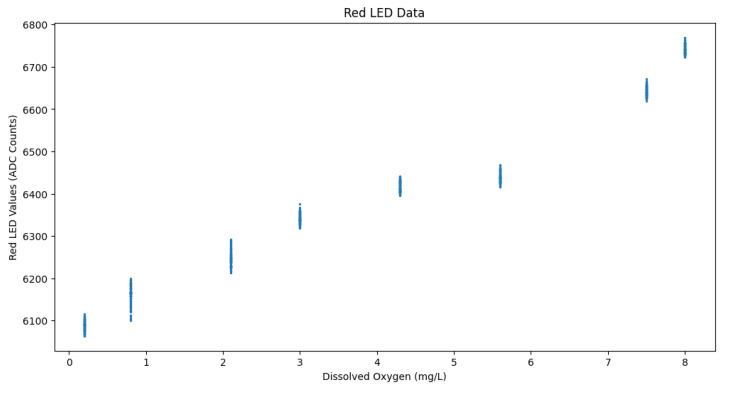
Scatter plot of Red LED data.

The compiled tabulated dataset includes data collected via MAX30102 sensor from multiple batches of samples at each DO level. The columns of the dataset consisted of the dissolved oxygen content, in mg/L obtained from the MW600 meter readings, the red LED reading from the MAX30102 sensor, and the infrared reading obtained from the MAX30102 sensor. The size of the dataset was 800 samples, comprising 100 readings from each of the DO levels. Upon plotting the relationship between the red LED readings and DO levels, it was found that, generally, as the red LED value increased so did the DO level, but in a step-wise fashion as can be seen in Figure 2. Since the DO values were fixed in the water samples, they are displayed on the X-axis and the amount of red LED light reflected and measured by the sensor is then depicted on the y-axis. Most importantly though, there were noticeable overlaps between the red LED values that had differing DO levels.

**Figure 3:**
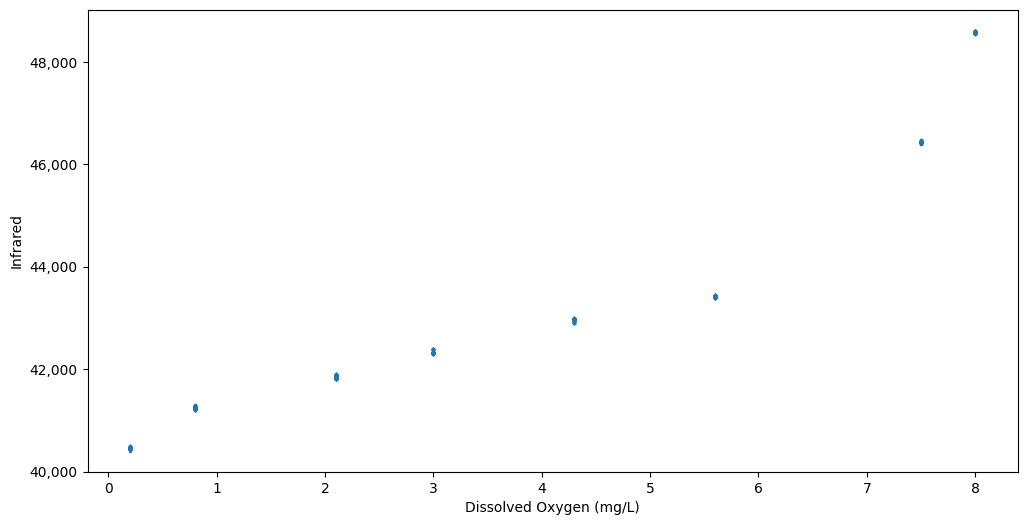
Scatter plot of infrared data.

Plotting the relationship between the infrared readings and DO levels revealed a similar trend where as the infrared values increased, the DO levels also increased in a step-wise fashion, but with no overlapping of the same DO levels having different infrared reflection levels, as portrayed in Figure 3. These results also correlate with and further support the findings of Miura et al. [15] who measured the absorbance of different wavelengths of light on 108 samples of sea water and tap water.

The dataset was then split into 70% for training and 30% for testing various machine learning regression models. The models used were sklearn’s linear regression [26], support vector machine regression (SVR) [27] with a radial basis function (RBF) kernel [28], and scipy’s orthogonal distance regression (ODR) [29]. The first two models were trained once with red LED data, once with the infrared data, and once with both red LED and infrared data, to determine which of the sensor data performs the best for converting to DO content. But since the Red LED Data had significant DO level overlaps, as can be seen in Figure 2, and the fact that Miura et al. [15] found that the infrared wavelength was one of the best light wavelengths for measuring DO absorption levels, the ODR model was trained on the infrared data only. A 10-fold cross validation was used on the training data for all of the regression models. The SciPy library’s curve_fit [30] was also utilized with quadratic, cubic, and quartic equations in an attempt to obtain an equation that could easily be visualized, unlike SVM with an RBF kernel.

### 4.3. Formulation and Results

**Figure 4:**
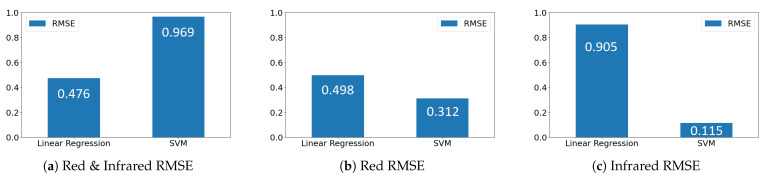
RMSE visualizations.

Upon analysis from the 10-fold cross-validation for the linear regression and SVM models, linear regression only outperformed the SVM model on the dataset using both red LED and infrared data, with a root mean squared error (RMSE) of 0.476, compared to SVM’s poor RMSE of 0.969, as depicted in Figure 4a. Figure 4b shows the RMSE on the solely red LED dataset, where linear regression and SVM performed decently having RMSEs of 0.498 and 0.312 respectively. The best-performing model was however derived from the infrared dataset, shown in Figure 4c, where linear regression performed poorly with an RMSE of 0.905 but SVM had an outstanding RMSE of 0.115. These results are also shown in tabular form in Table 1 for easier comparison.

The 10-fold cross validation results for the ODR regression model was also carried out on the infrared dataset, and takes into account the existence of errors across both the x and y variables. The cross validation was run across linear, cubic, quartic, and sigmoidal functions. The linear function had an average RMSE of 0.909 and an average Orthogonal Distance Error (ODE) of 0.7827. The cubic function had an average RMSE of 0.389 and an average ODE of 0.324, while the quartic function had an average RMSE of 0.389 and an average ODE of 0.331. The sigmoidal function had an average RMSE of 0.186 and an average ODE of 0.174. Overall, the best performance here was produced by the sigmoidal function.

For the curve_fit functions, as expected, the higher degree functions had a lower RMSE and higher R^2^ The quadratic, cubic, quartic, and sigmoidal functions had RMSE of 0.3954, 0.384, 0.111, and 0.186, and R^2^ scores of 0.979, 0.980, 0.998, and 0.995 respectively. While the quartic function performs well, it likely would not generalize well to outside data as its function likely overfits to the training dataset used here. It would be more practical to use the quadratic or cubic functions as they would likely generalize better to outside data.

**Figure 5:**
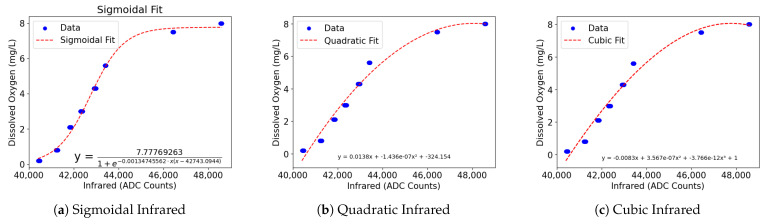
ODR visualizations.

The Orthogonal Distance Regression (ODR), which also provides fit graphs, had some differences from the curve_fit function. ODR provides its goodness of fit using residual variance, where values closer to 0 indicate a better fit and a value of 0 indicates a perfect fit. Both the residual variance and the R^2^ values are provided here and in Figure 5 to provide additional context. For the quadratic function, an RMSE of 0.395, an R^2^ value of 0.979 and a residual variance of 0.1596 was observed. For the cubic function, an RMSE of 0.388, an R^2^ of 0.98799, and a residual variance of 0.155 were observed. For the sigmoidal (logistic) function, an RMSE of 0.979, an R^2^ of 0.995, and a residual variance of 0.030 were observed.

The curve was only fit up until 8 mg/L, which is sufficient for interpolation of DO readings in the existing test-bed in the research lab on campus as well as in aquaculture settings. The first real world application of DOxy is the fish farming industry where the concentration of 5 mg/L DO is recommended for optimum fish health. Overall, most species of fish are at risk when DO levels fall to around 2–4 mg/L [31]. The United States Environmental Protection Agency (EPA) generally consider DO levels below 3 mg/L as inhabitable environments for fish and DO levels below 1 mg/L as “hypoxic and usually devoid of life” [32] a.k.a. Dead zones [33].

## 5. DOxy Meter

Using the aforementioned derived formulas, an IoT meter named DOxy (short for Dissolved Oxygen) was constructed and tested in both the lab and real world settings. DOxy leverages the quenching effect that dissolved oxygen has on the fluorescence of a beam of light fired at water, an approach that allows DOxy’s Sensing Units (SU)s to be small and cost-effective as well as passive with respect to observed systems, promoting its long-term deployment.

### 5.1. Electronics

**Figure 6:**
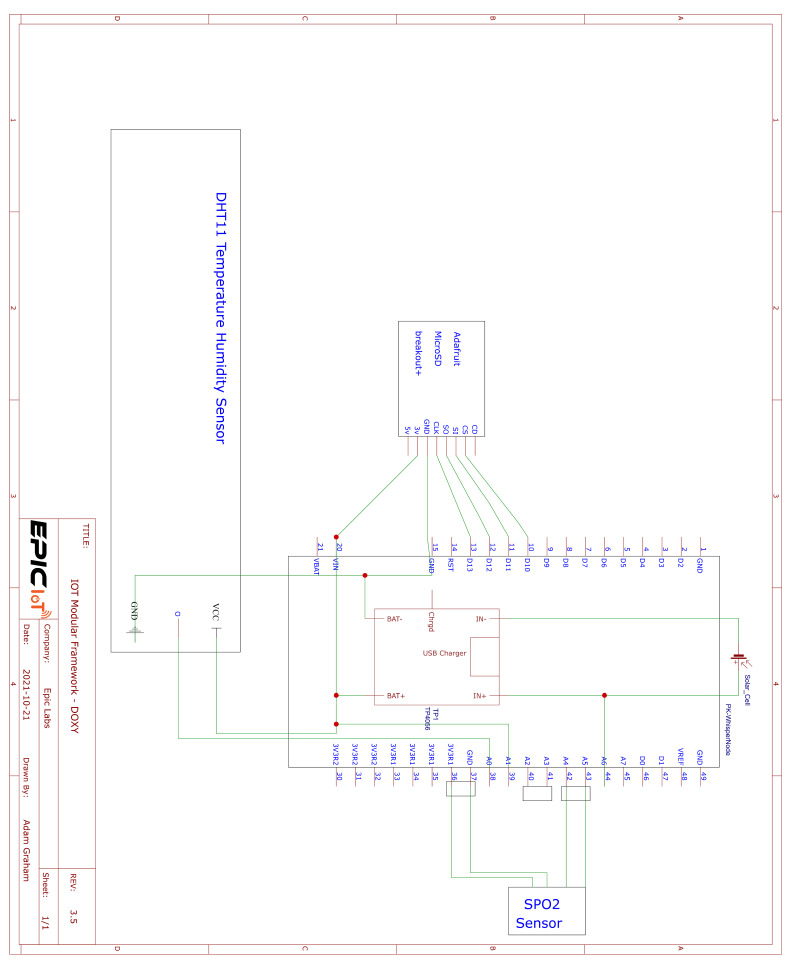
Standalone DOxy schematic.

DOxy was created in tandem with the Modular IOT Platform reported on in [34]. There are two versions of DOxy’s electronics, one that can be used as a standalone product and the other that can be plugged into existing products. The standalone version is powered by a Wisen Whisper Node [35] micro-controller with an on-board LoRa (Long Range) [36] communication module [37] with 3DBI Omni-directional long range external antenna [38], a DHT11 Temperature and Humidity Sensor [39], a TP4056 battery charge controller [40], a 5 W lithium battery pack, and a 5 V 100 mAh solar panel. Using a solar panel makes the system more flexible with regards to where it can be deployed, as it does not require hard-lined power in order to work. The standalone version contains a MicroSD card adapter module [41] to enable local storage of data and resilience in the case of network or even device failure. This also allows for storage of things like a device ID or other information about the system. A schematic of the standalone hardware can be seen in Figure 6.

**Figure 7:**
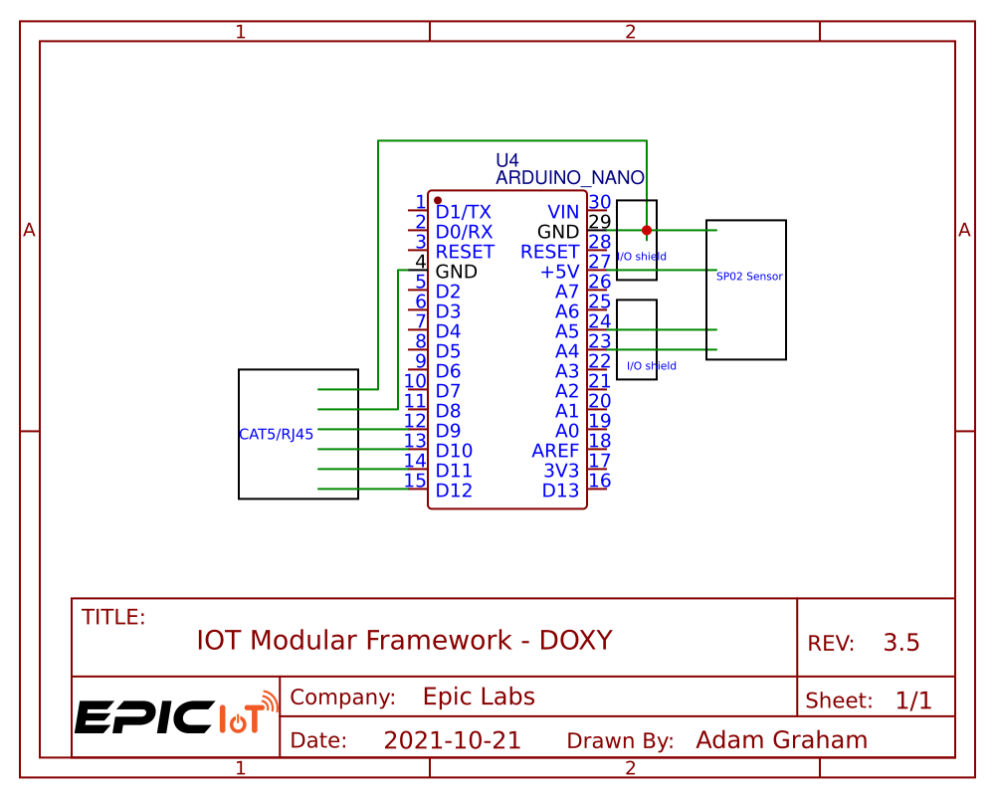
Plug and Play DOxy schematic.

The Plug and Play (PnP) version is similar to the standalone version, but instead is powered by a Arduino Nano micro-controller [42] and connects to the host device using a Cat-6 cable with an RJ45 Connector. The PnP version does not contain a battery module nor an ambient temperature sensor. A schematic of the PnP hardware can be seen in Figure 7.

### 5.2. 3D Printed Casing

**Figure 8:**
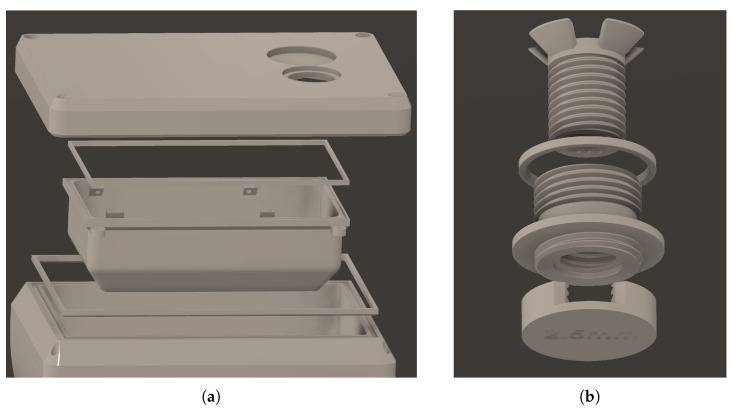
3D-printed casing: (**a**) top section (W158 mm × D108 mm × H112 mm); (**b**) lens housing (D35 mm × H46.6 mm).

The DOxy device is housed in a PolyEthylene Terephthalate Glycol-modified (PETG) [43,44,45] 3D printed casing prototyped and tested first in [7] and then enhanced as shown in Figure 8a, which keeps the device buoyant and waterproof by utilizing the best practices described in [46]. A primary gasket also 3D printed in PETG is used to waterproof the connection between the case’s lid and main body. The hole in the top of the case’s lid holds a cable gland for the communication and power cables that connect to the antenna and solar panel respectively. The section under the lid is a specialized compartment that houses the PCB and waterproofs it with another gasket that connects it with the lid of the case. A secondary cable gland protrudes from the bottom of the PCB compartment to waterproof the sensor cable and direct it into the sensor housing.

The sensor housing consists of three main sections as shown in Figure 8b. The first section holds the sensor, with 3 small screw holes on the bottom to ensure the sensor is firmly secured. The sensor holder is then threaded into the plexiglass lens housing to ensure the sensor is completely parallel to the lens. The plexiglass and plexiglass lens housing are needed in order to ensure the separation of the sensor’s electronics from the water and thus avoiding damage to the sensor by keeping it dry at all times. The lens housing has the lens inserted at the very bottom where the threading ends internally. A gasket is inserted into a rim near the bottom of the lens housing to ensure the sensor assembly is waterproofed. And the final section provides a backdrop for the light wave from the sensor to reflect off of and return to the sensor’s collector. This backdrop section is screwed on to the external threading of the lens housing and has large opening along three of its sides in order to ensure no air bubbles get caught in between the lens and the backdrop as that would effect the refraction of the emitted light by the sensor.

### 5.3. Communication

The standalone DOxy system SUs are capable of transmitting their measurements to the web based dashboard in several different ways which is dependent on the environment they are being set up in.

#### 5.3.1. Single-Hop Communications

For instance, if they are set up in a facility with ample WiFi availability or in the back yard of a home hydroponic system within the home WiFi signal range, then the SUs can use an ESP8266-01 WiFi module [47] to send a POST request containing JSON sensor data to the web server through the internet. But if for instance the SUs are in a remote location where maybe only Cellphone service is available, then they can be quipped to utilize the GSM network instead.

#### 5.3.2. Multi-Hop Communication

For bigger or more remote operations where WiFi and/or GSM are not available or suitable, the SUs could send data to a Base Station (BS) responsible for compiling various sensor data from multiple SUs in one or several sites and relaying this information to the web dashboard, and/or handling any actuation capabilities the system may be configured to perform given the various possible DO level readings. In this scenario, numerous wireless communication technology can be utilized between the SUs and the BS such as Zigbee, nrf, LoRa, or even WiFi based on their distance, line of site, and other environmental factors.

For handling this type of communication, the DOxy system uses an in house built Energy Aware Communication Protocol (EACP) named Âb [48]. ÂB is a responsibility protocol which sits between layers 2 and 3 of the TCP-IP communication stack and thus provides layer 2 agnostic end-to-end communication capable of utilizing the low energy sleep mode of all the network hops and not only the initial transmitter and/or final receiver of the data packets. ÂB is currently in use in the Hydration Automation (HA) system [49] and Smart Tanks [50]. where LoRA is utilized to transmit the water level of water tanks in use for agricultural purposes to a pumping station; as well as in another implementation of DOxy in both a deeply forested area necessitating many short range Zigbee hops from a river in Malaysia to a near by research lab at a school [51] and a Remotely Operated Vehicle (ROV) in a lake communicating to the shore and then from there to a research facility using the Message Queue Telemetry Transport (MQTT) protocol [52]. These two usages of DOxy and Âb in Malaysia have thus provided international specifications and considerations to the system.

### 5.4. Web Based User Dashboard

In order to effectively collect and present data from DOxy SUs, a comprehensive dashboard system was developed. The dashboard is composed of a data access layer (backend) composed of a database and an Application Programming Interface (API) for posting/fetching values to/from the database, along with a presentation layer (frontend) Graphical User Interface (GUI). The SUs report their measurements to a base station, which then makes an HTTP request to the backend API in intervals configurable by the user. The various sensor readings are stored in the database and the frontend allows for easy visualization and analysis of that data with clear and concise graphs and charts.

#### 5.4.1. Tech Stack: Data Access Layer (Backend)

The backend API is built using Node.js [53] which is a popular and efficient JavaScript platform for building server-side applications. Node.js allows for fast and scalable network applications, making it an ideal choice for DOxy and IoT systems in general. The backend of the system is launched as multiple independently maintainable and expandable microservices using Docker [54] in order to add flexibility and scalability to the system. To facilitate communication between the sensors or the base station, and the dashboard, the backend uses both an HTTP request and a WebSocket. The HTTP protocol allows for the transmission of data between the backend and frontend, while the WebSocket connection allows for live data display and receiving information from the base station.

The database used in the system is TimescaleDB [55], which is a time-series database optimized for storing and querying large amounts of time-stamped data. It is built on top of PostgreSQL and can handle high read and write loads, making it well-suited for storing data from IoT sensors that generate large amounts of data over time. The database consists of tables with user IDs that store sensor data for each user, with a schema for each type of sensor (DO, temperature, etc.). The schema defines the structure of the data stored in the table, including the names and data types of the columns, as well as any constraints or indexes on the data. By using a schema for each type of sensor, we can ensure that the data from each sensor is stored in a consistent and organized manner. The database is pseudo-schema-less in that an external command-line interface (CLI) tool built with Rust is used to add new schemas to the database when new sensors (such as for measuring atmospheric pressure—as discussed in Section 7) are added to the system. This means that new sensors can be added to the system without having to modify the underlying database schema, which is a more flexible and scalable approach.

The backend service was deployed on an Amazon Web Services (AWS) EC2 instance as a Docker container, orchestrated by Docker Compose. EC2 is a cloud computing service offered by AWS that allows users to run applications on virtual machines in the cloud in order to abstract away all of the setup and maintenance needs for the backend environment, and docker Compose is a tool that allows the definition and execution of multi-container Docker applications, making it easier to manage and deploy the service.

#### 5.4.2. Tech Stack: Presentation Layer (Frontend)

For the frontend, the svelte [56] JavaScript web framework was utilized, along with Chart.js [57], in order to create a user-friendly interface for displaying sensor readings. Svelte is a modern framework that provides an efficient and reactive way to build user interfaces with responsive layouts, allowing for seamless use on various devices with various screen sizes. Chart.js is a powerful JavaScript library that allows for the creation of visually appealing and interactive charts, allowing users to easily analyze the data from the sensors. Technically though, Svelte has a Server-Side Renderer (SRR) which is set up as another microservice in the backend. SRR is a technique that renders the generated HTML on the server rather than in the client’s browser which improves the performance of the web application, as the rendered HTML can be sent to the client faster than the client’s browser could render the HTML code itself.

**Figure 9:**
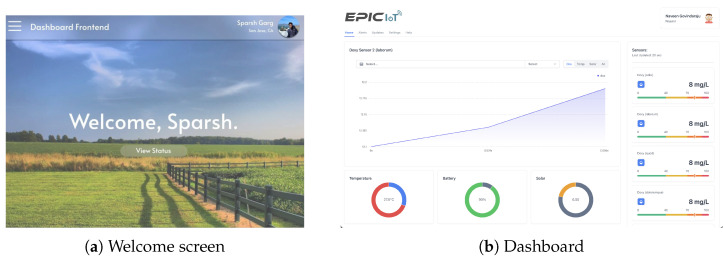
Dashboard GUI.

The authentication system supporting login functionalities with Google accounts as well as phone number login were added in order to separate and secure user data. When a user logs in with their Google account or phone number, the system verifies their identity and grants them access to the dashboard based on their credentials. Figure 9a shows an example of the post authentication welcome screen. The dashboard also allows for easy navigation and filtering of data, allowing users to focus on specific aspects of the data. For instance, the user can navigate to any of their sensors in order to see data from the sensors displayed in clear and concise graphs and charts as depicted in Figure 9b. Each sensor object on the dashboard has a set of instructions on how to display its data. For example, a sensor may display water oxygen levels as a line chart when in focus, and as a pie chart with a percentage when minimized. This allows for the customization of the visual representation of data based on the specific needs and characteristics of each sensor.

## 6. Results

Through a number of parallel readings of DO levels in water samples by both DOxy’s High-sensitivity Pulse Oximeter and a standard dissolved oxygen meter, two approaches were investigated: One, in which various machine learning models were trained and tested to produce a dynamic mapping of sensor readings to actual DO values in the lab as discussed in Section 4.3 in detail. And another in which curve-fitting models were used to find the best fitting curve for producing a successful conversion formula that was then programmed into the DOxy SUs to be used offline. The DOxy SUs were repeatedly tested in lab produced beakers of carefully controlled water samples as well as buckets filled with tap water, random water fountains on Santa Clara University’s main campus, and a nearby lake, all with accurate DO readings as confirmed with parallel readings from the Milwaukee MW600 DO meter. Table 2 lists the side by side results of measurements of DO in water samples with the same DO levels by the commercial Milwaukee MW600 DO meter and the DOxy sensing unit equipped with the Maxim Integrated MAX30102 Oximeter sensor.

**Figure 10:**
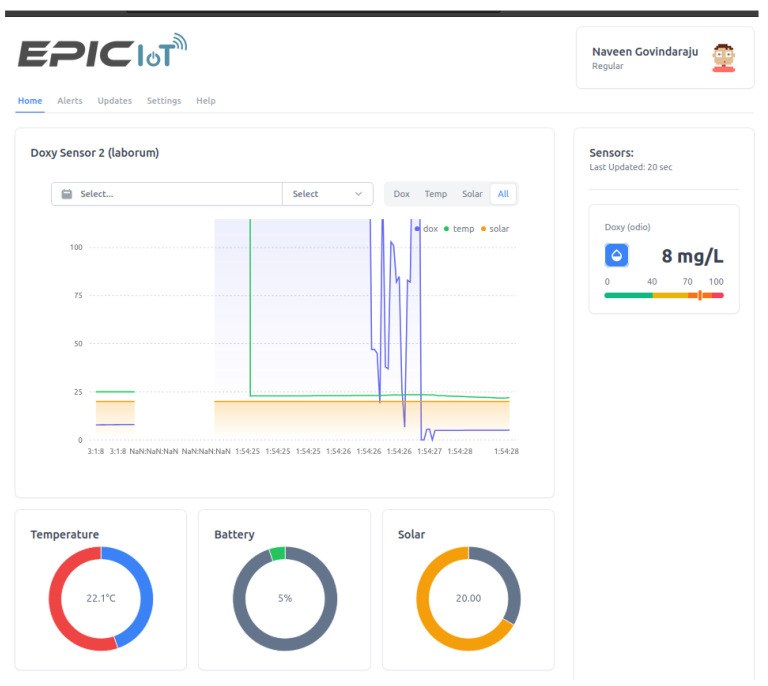
DOxy results displayed on the dashboard.

In all cases, the sensor oscillated and then settled down on the correct reading once in the water for a few seconds. As an example, Figure 10 shows the oscillation and subsequent settling down on the constant reading of 8 mg/L of DO when the SU was placed in one of the water fountains on the SCU campus.

## 7. Work in Progress

Several theoretical and technical directions for improving measurements are under exploration and development as reported on in this section.

### 7.1. Effects of Temperature on DO Measurements

**Figure 11:**
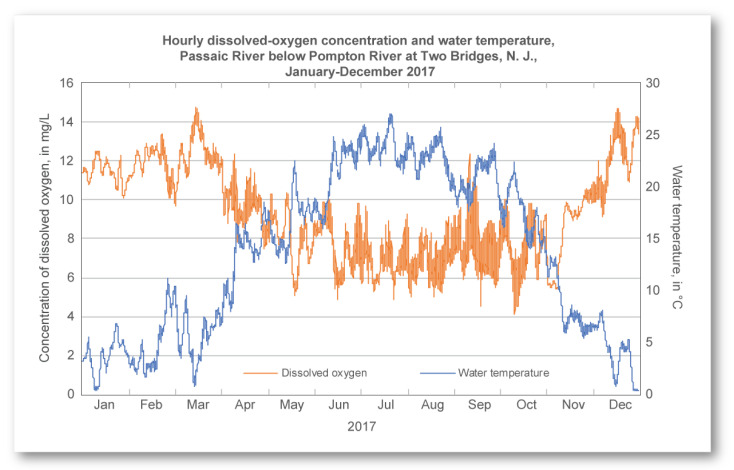
USGS Sample chart showing the effect of temperature on dissolved oxygen concentration in a body of water [33].

Variance in temperature can effect the concentration of dissolved oxygen in water [33]. The colder the water is the higher concentration of dissolved oxygen it can hold [33] thus the temperature and dissolved oxygen concentration are inversely proportioned. As displayed in Figure 11 there is a significant drop of concentration of dissolved oxygen between colder months and warmer months.

Because the data collection performed to build the machine learning models used in DOxy was done at room temperature (20–22 °C), it is necessary to account for the temperature variation when applying the models to real-world scenarios. By understanding the relationship between temperature and dissolved oxygen concentration, the measurements can be adjusted in order to reflect the conditions of the surrounding environment.

To convert the room temperature DO measurement to the DO concentration at the surrounding temperature, an oxygen solubility chart can be utilized in order to supply the solubilities for the van’t Hoff equation [58]. The van’t Hoff Equation (2) is a thermodynamic equation that relates the solubility of a gas in a solvent to temperature. It provides an approximation of how the solubility of a gas changes with temperature under certain assumptions. In the context of dissolved oxygen in water, the van’t Hoff equation can be used to estimate the change in solubility of oxygen as the temperature changes. The equation allows the calculation of a scaling factor that represents the relative change in solubility between two temperatures.

(2) $$\ln\left(\frac{S_{2}}{S_{1}}\right)=\frac{\Delta H}{R}\left(\frac{1}{T_{1}}-\frac{1}{T_{2}}\right)$$

where:

- S1 is the solubility of oxygen at temperature $T_{1}$;
- S2 is the solubility of oxygen at temperature $T_{2}$;
- ΔH is the heat of solution (usually a constant for a particular gas);
- *R* is the gas constant;
- $T_{1}$ and $T_{2}$ are the respective temperatures in Kelvin.

To scale the dissolved oxygen values from room temperature ($T_{1}$) to the desired temperature ($T_{2}$), a scaling factor can be calculated by isolating the solubility of oxygen at the two temperatures and multiplying it into the room temperature DO values in order to obtain the adjusted DO values for the desired temperature. The scaling factor can be calculated by isolating $\frac{S_{1}}{S_{2}}$ from Equation (2) as seen in Equation (3), then it is plugged into Equation (4) to obtain the final DO value.

(3) $$\frac{S_{2}}{S_{1}}=e^{\left(\frac{\Delta H}{R}\right)\left(\frac{1}{T_{1}}-\frac{1}{T_{2}}\right)}$$

(4) AdjustedDOvalues=RoomtemperatureDOvalues×scalingfactor

### 7.2. Effects of Atmospheric Pressure on DO Measurements

Atmospheric Pressure can effect the concentration of dissolved oxygen in water. The calculation for the concentration after taking atmospheric pressure into account can be done by using Henry’s law [59]. The dissolved oxygen concentration is proportional to the percent of oxygen in the air above it as can be seen in Equation (5), where *C* is the scaled concentration of dissolved oxygen in water, *k* is Henry’s law constant, and *P* is the percent of oxygen in the air above the water.

(5) C=k·P

There are two ways the scaling of the DO concentration value according to the atmospheric pressure can be accommodated for within the DOxy system: Either an additional atmospheric pressure sensor can be added to the DOxy SUs, or public API’s of atmospheric pressure data can be used to perform the calculations within the dashboard’s backend once the sensor readings have been received.

### 7.3. Sensor Enhancement

As the present research has shown, using purely the infrared data from the MAX30102 sensor, the DO content in water can be measured accurately. Which is in line with, the findings of Miura et al. [15] that shows the greatest variance in absorbance occurs in the blue and infrared light wavelengths in water. Wibowo et al. however, set up a fluorescence-quenching dissolved oxygen sensor, running it first with a red LED and then with a blue LED, finding that a blue light was more sensitive to dissolved oxygen levels [60]. Hence, for future implementation, research is being conducted on the effectiveness of the blue light for measuring dissolved oxygen in water both by itself, and in combination with data from infrared light. Based on those results, the sensor used in DOxy SUs may change or maybe even a completely new sensor is developed.

### 7.4. Range Extension

Since the measurement of DO is not a practice limited to fish farming alone, the range of DOxy will be extended well beyond its current rage. DOxy will be able to be utilized to measure DO levels in bodies of water for environmental purposes such as overall water quality testing (usually along with temperature, ph levels, and other metrics), discovering dead zones (DO levels below 1 mg/L) [33], and determining if it is healthy for human consumption (DO levels above 6.5–8 mg/L) [61,62] as well as how it will taste.

### 7.5. Actuation

Without loss of generality, it can be seen that a DOxy sensor can be used to control various environmental factors in an aquaculture setup. For instance, if the measured level of dissolved oxygen falls below a set certain level in a tank, the DOxy sensor readings could trigger the opening of oxygen valves or even the turning on of oxygen pumps that push more oxygen into the water until the DOxy readings show a restoration of the DO levels.

## 8. Conclusions

Measuring dissolved oxygen levels in water is achievable with use of none chemical based optical sensors. However, most current work in this area is academic and not market-ready. DOxy is a low-cost, accessible, and sustainable optical DO metering system which will transform the aquaculture industry in this respect. Given the importance of monitoring DO levels, an IoT solution such as DOxy has the potential to reduce labor and thus improve the efficiency and productivity of aquaculture via automation. A strong correlation between values produced by DOxy and values produced by a DO meter show the viability and accuracy of DOxy’s approach at measuring DO in water. Furthermore, DOxy utilizes a web-based user-friendly dashboard to help users effectively visualize and analyze the data collected from sensors in the field. But most importantly, such aquacultural automation allows for early detection of changing water conditions and hence increases the quality of life for marine life as well as those who care for or whose livelihood depends on marine life.

## Figures and Tables

**Table 1:** Linear regression vs. SVM RMSE values for red and infrared data.

Red/Infrared Data	Linear Regression	SVM
Red and Infrared	0.476	0.969
Red	0.498	0.312
Infrared	0.905	0.115

**Table 2:** Side-by-side readings from Milwaukee MW600 meter and DOxy sensing unit.

Meter Reading	DOxy Reading
7.7	7.723882
7.7	7.725791
7.7	7.724597
7.7	7.725791
7.7	7.723737
7.7	7.725791
7.7	7.722496
7.7	7.724383
7.7	7.723665
7.7	7.72381
5.6	5.582961
5.6	5.55312
5.6	5.595675
5.6	5.546694
5.6	5.59779
5.6	5.51872
5.6	5.589324
5.6	5.527349
5.6	5.589324
5.6	5.512235
0.3	0.333782
0.3	0.320689
0.3	0.333782
0.3	0.332493
0.3	0.355979
0.3	0.337677
0.3	0.355522
0.3	0.356437
0.3	0.359197
0.3	0.346044

## Data Availability

Research data can be shared by Authors if requested.
